# The SARS-CoV-2 B.1.351 Variant Can Transmit in Rats But Not in Mice

**DOI:** 10.3389/fimmu.2022.869809

**Published:** 2022-04-28

**Authors:** Cheng Zhang, Huan Cui, Entao Li, Zhendong Guo, Tiecheng Wang, Fang Yan, Lina Liu, Yuanguo Li, Di Chen, Keyin Meng, Nan Li, Chengfeng Qin, Juxiang Liu, Yuwei Gao, Chunmao Zhang

**Affiliations:** ^1^ Changchun Veterinary Research Institute, Chinese Academy of Agricultural Sciences, Changchun, China; ^2^ College of Veterinary Medicine, Hebei Agricultural University, Baoding, China; ^3^ College of Veterinary Medicine, Jilin University, Changchun, China; ^4^ Beijing Institute of Microbiology and Epidemiology, Beijing, China

**Keywords:** SARS-CoV-2, B.1.351, contact transmission, mice, rats

## Abstract

Previous studies have shown that B.1.351 and other variants have extended the host range of severe acute respiratory syndrome coronavirus 2 (SARS-CoV-2) to mice. Sustained transmission is a prerequisite for viral maintenance in a population. However, no evidence of natural transmission of SARS-CoV-2 in wild mice has been documented to date. Here, we evaluated the replication and contact transmission of the B.1.351 variant in mice and rats. The B.1.351 variant could infect and replicate efficiently in the airways of mice and rats. Furthermore, the B.1.351 variant could not be transmitted in BALB/c or C57BL/6 mice but could be transmitted with moderate efficiency in rats by direct contact. Additionally, the B.1.351 variant did not transmit from inoculated Syrian hamsters to BALB/c mice. Moreover, the mouse-adapted SARS-CoV-2 strain C57MA14 did not transmit in mice. In summary, the risk of B.1.351 variant transmission in mice is extremely low, but the transmission risk in rats should not be neglected. We should pay more attention to the potential natural transmission of SARS-CoV-2 variants in rats and their possible spillback to humans.

## Introduction

Severe acute respiratory syndrome coronavirus 2 (SARS-CoV-2) has spread worldwide and severely disrupted the healthcare system and socioeconomic activities. As a single-stranded positive RNA virus, SARS-CoV-2 has undergone frequent mutations during global transmission, which has led to the emergence of several important variants of concern (VOCs), such as B.1.1.7 (alpha), B.1.351 (beta), P.1 (gamma), B.1.617.2 (delta) and B.1.1.529 (omicron) (1, 2).

The spike protein of SARS-CoV-2 mediates viral cell entry by binding to the cellular receptor ACE2 (3). Several prevalent mutations have been identified in the receptor-binding domain (RBD) of the spike protein (4, 5), which may extend the host range or switch host tropism. Previous studies showed that N501Y alone (6, 7) or in combination with K417N/T and E484K enhanced the affinities to mouse (4, 7–9) and rat ACE2 proteins (10) and conferred the SARS-CoV-2 variants the ability to infect mouse cell lines (6, 11). Furthermore, Xavier Montagutelli et al. showed that B.1.351 and P.1 variants containing the N501Y, K417T/N and E484K mutations had an extended host range that included mice (11, 12), and Qi Chen et al. showed that the B.1.351 variant infected aged mice and replicated efficiently in the airways of mice (13). Surprisingly, these mutations were also found in three mouse-adapted SARS-CoV-2 strains, MASCp36, MA-SARS-CoV-2 and MA30 (14–16).

Given that mice and other animals of the family Muridae live in proximity to humans, these results suggest the risk of mice or other animals of the family Muridae serving as new reservoirs for SARS-CoV-2 variants in regions where the B.1.351, P.1 or other emerging variants are prevalent and from where they might evolve independently and possibly transmit back to humans (17). Sustained transmission is a prerequisite for viral maintenance in a population or as a reservoir. However, no evidence of natural transmission of B.1.351 and P.1 in wild mice has been documented to date. To determine the potential transmission of SARS-CoV-2 variants in animals of the family Muridae, we evaluated the replication and direct contact transmission of the B.1.351 variant in mice and rats. The experimental results showed that the B.1.351 variant replicated efficiently in the airways of mice and rats. Moreover, the B.1.351 variant did not transmit in mice but could transmit with moderate efficiency in rats by direct contact.

## Results

### The B.1.351 Variant Replicated Efficiently in the Airways of Mice and Rats

**Figure 1:**
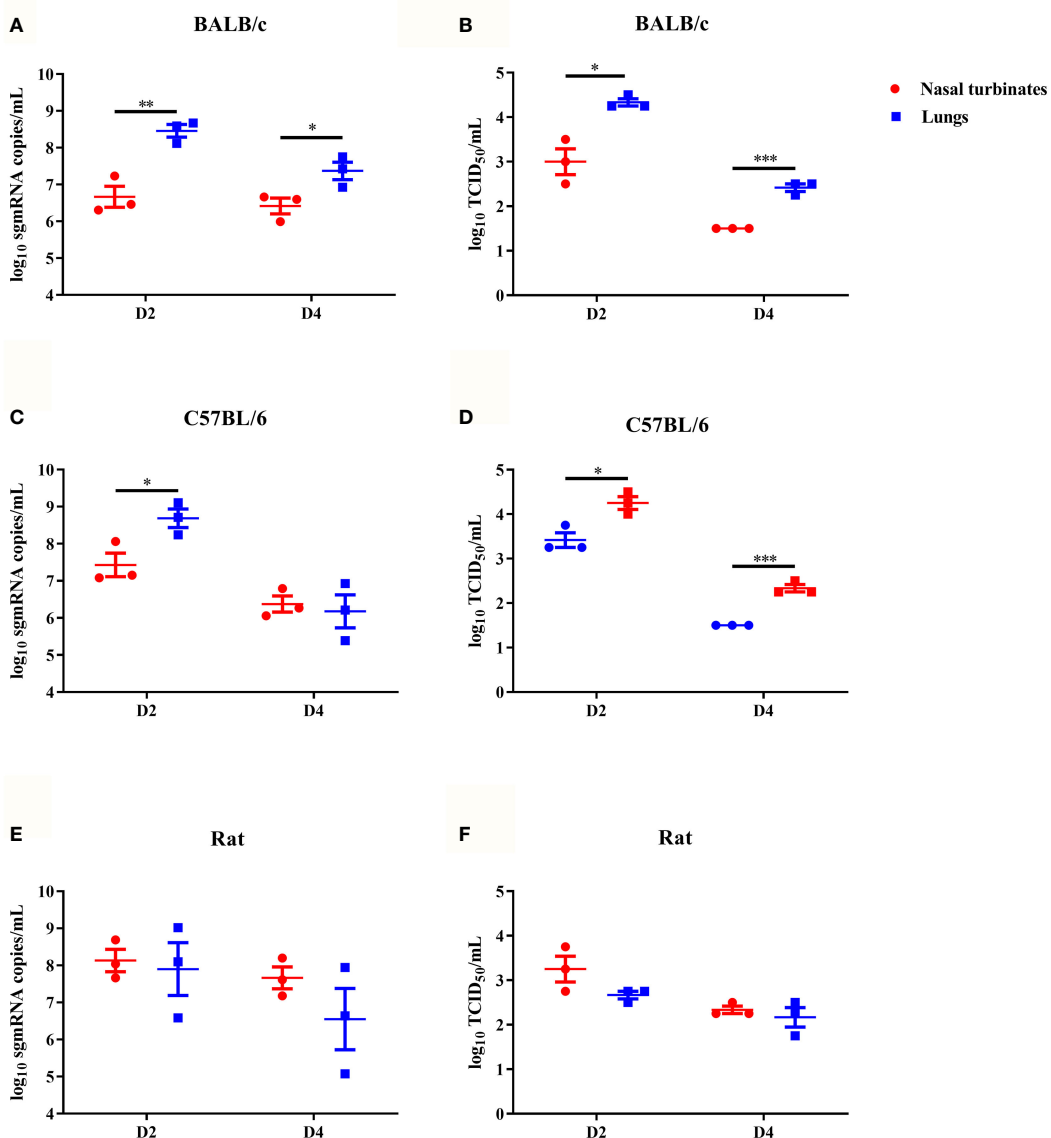
The SARS-CoV-2 B.1.351 variant replicated efficiently in mice and rats. The dotted line represents the detection limit. Viral subgenomic RNA (sgRNA) load (log_10_ sgRNA copies/mL) in the nasal turbinates and lungs of inoculated female BALB/c mice **(A)**, female C57BL/6 mice **(C)** and male rats **(E)**. Viral titres (log_10_ TCID_50_/mL) in the nasal turbinates and lungs of inoculated female BALB/c mice **(B)**, female C57BL/6 mice **(D)** and male rats **(F)**. All data are represented as the mean ± sem, and the number of replicate animals in each group was 3 (n=3). An unpaired t test was used to analyze the significant differences in viral titres and viral sgRNA loads between nasal turbinates and lungs (p<0.05,*; p<0.01,**; p<0.001,***).

To characterize viral replication in mice, six-week-old female BALB/c and C57BL/6 mice were intranasally inoculated with the B.1.351 variant on day 0 (D0), and then the viral subgenomic RNA (sgRNA) loads and viral titres in the nasal turbinates and lungs on D2 and D4 were determined. High viral sgRNA loads and viral titres were detected in the lungs of the two mouse strains on D2, which then rapidly decreased on D4 (**Figures 1A–D**). Compared with those in the lungs, the viral sgRNA load and viral titre in the nasal turbinates were lower on D2, and the viral titres in nasal turbinates rapidly decreased to an undetectable level in both mouse strains on D4 (**Figures 1A–D**). We also characterized the viral replication dynamics in rats. The B.1.351 variant moderately replicated in the lungs and nasal turbinates of rats on D2, and the load gradually decreased on D4 (**Figures 1E, F**). In summary, the B.1.351 variant could infect and replicate efficiently in the airways of mice and rats.

### The B.1.351 Variant Did Not Transmit in BALB/c and C57BL/6 Mice

**Figure 2:**
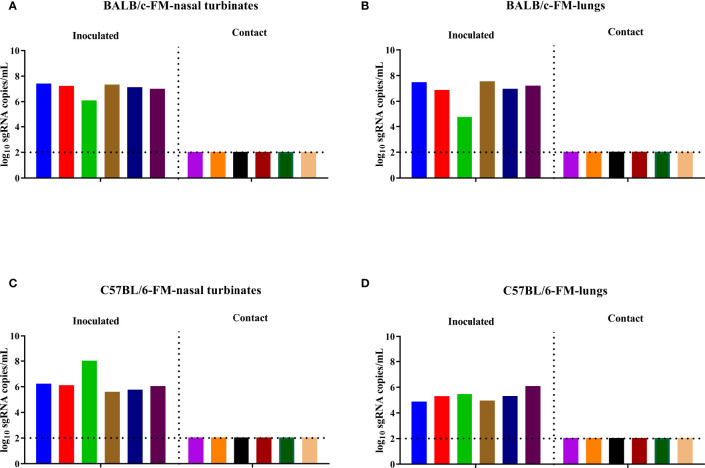
SARS-CoV-2 B.1.351 variant transmission in female BALB/c and female C57BL/6 mice. The dotted line represents the detection limit. Viral sgRNA copies (log_10_ sgRNA copies/mL) in the nasal turbinates **(A)** and lungs **(B)** of the inoculated female BALB/c mice and the contact female BALB/c mice. Viral sgRNA copies in the nasal turbinates **(C)** and lungs **(D)** of inoculated female C57BL/6 mice and contact female C57BL/6 mice.

To evaluate the direct contact transmission of the B.1.351 variant in mice, six female BALB/c mice were inoculated with the virus on D0 and cohoused with another six naïve female BALB/c mice on D1. The inoculated BALB/c mice and the contact BALB/c mice were sacrificed to collect nasal turbinates and lungs on D4 and D5, respectively, and then the viral sgRNA load in the nasal turbinates and lungs was determined. A high level of viral sgRNA was detected in the nasal turbinates and lungs of all inoculated female BALB/c mice, while no viral sgRNA was detected in the nasal turbinates and lungs of the contact female BALB/c mice (**Figures 2A, B**). We also performed direct contact transmission experiments with female C57BL/6 mice. Similar to the results of transmission experiments in female BALB/c mice, a high level of viral sgRNA was detected in the nasal turbinates and lungs of the inoculated female C57BL/6 mice, but no viral sgRNA was detected in the nasal turbinates and lungs of the contact female C57BL/6 mice (**Figures 2C, D**). To determine the effect of sex differences on the contact transmission of the B.1.351 variant in mice, we performed contact transmission experiments with male BALB/c and male C57BL/6 mice. The B.1.351 variant replicated well in the airways of the inoculated male BALB/c and male C57BL/6 mice, but no viral sgRNA was detected in the contact male BALB/c and male C57BL/6 mice (**Supplementary Figures 1A–D**). In summary, the transmission experimental results showed that the B.1.351 variant could not transmit in mice.

### The B.1.351 Variant Could Transmit in Rats by Direct Contact

**Figure 3:**
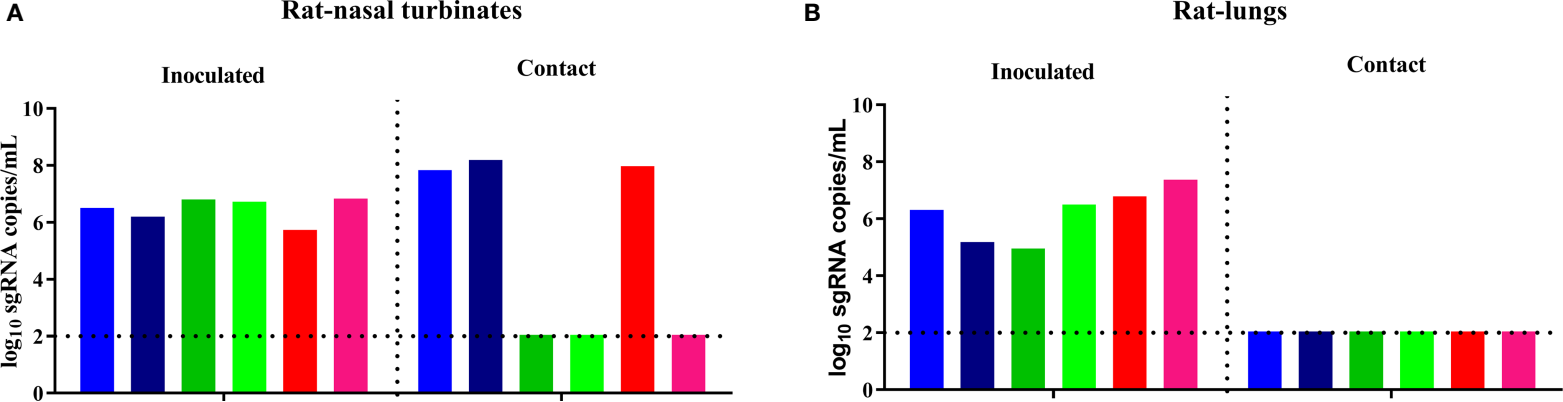
SARS-CoV-2 B.1.351 variant transmission in male rats. The dotted line represents the detection limit. Viral sgRNA copies (log_10_ sgRNA copies/mL) in the nasal turbinates **(A)** and lungs **(B)** of the inoculated rats and the contact rats. Every two rats in each of the three subgroups are shown in similar colors, such as red, blue and orange.

To assess the potential contact transmission of the B.1.351 variant in rats, three groups of two eight-week-old male rats were inoculated with the virus on D0, paired and cohoused together with another three groups of two naïve male rats (donor-contact ratio: 2:2) separately on D1. Viral sgRNA in the nasal turbinates and lungs of rats in the inoculated donor group and the contact transmission group was quantified on D4 and D5, respectively. A high level of viral sgRNA was detected in the nasal turbinates and lungs of the inoculated rats, and a high level of viral sgRNA was also detected in the nasal turbinates of three of the six contact rats, but no viral sgRNA was detected in the lung of any rat in the contact transmission group (**Figures 3A, B**). The viral transmission efficiency varied greatly in the different subgroups, with efficiencies of 100%, 0% and 50%. Hence, the experimental results showed that the B.1.351 variant had moderate contact transmission ability in rats.

### The B.1.351 Variant Did Not Transmit Between Syrian Hamsters and Mice

**Figure 4:**
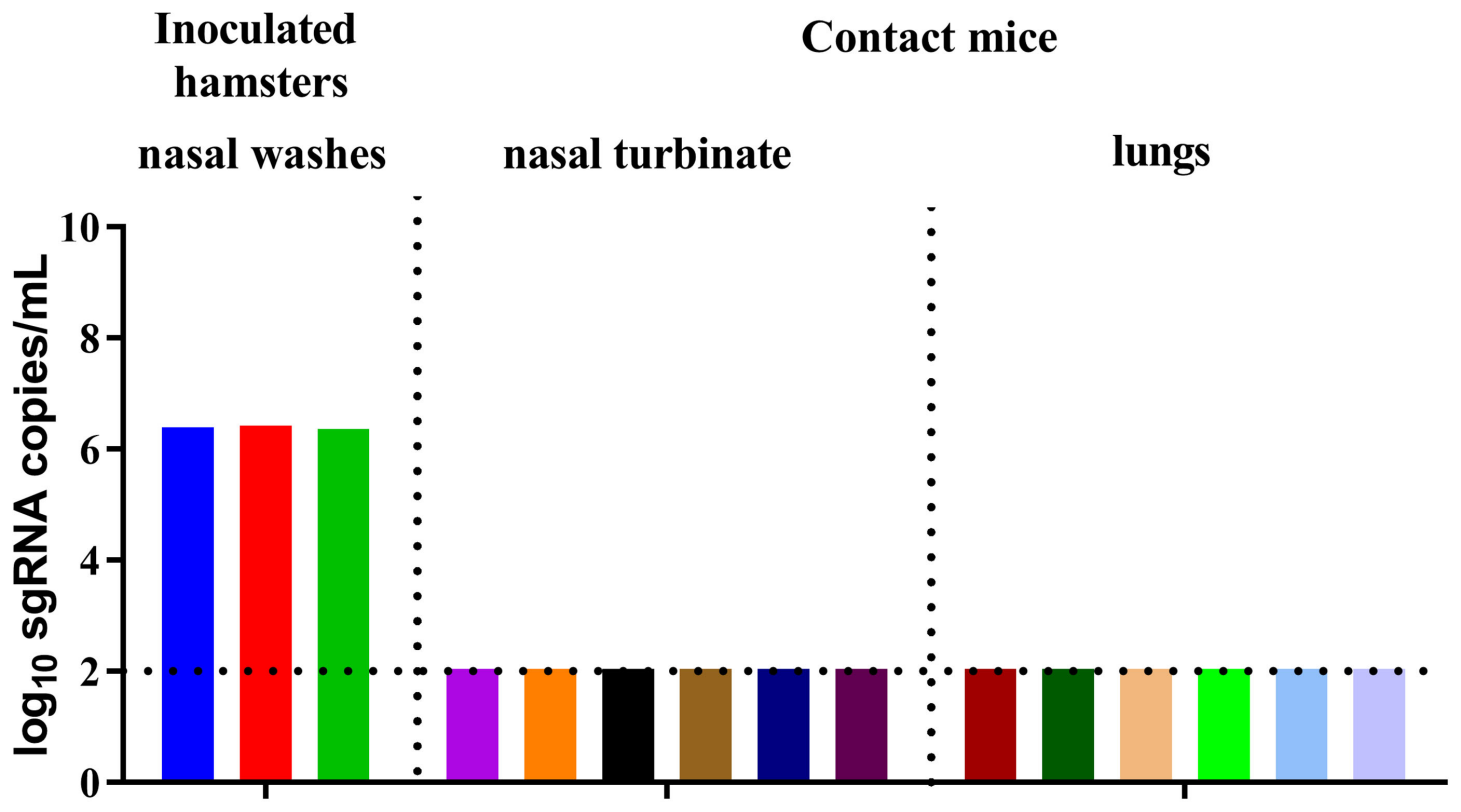
SARS-CoV-2 B.1.351 variant transmission between male Syrian hamsters and female BALB/c mice. The dotted line represents the detection limit. Viral sgRNA copies (log_10_ sgRNA copies/mL) in the nasal washes of the inoculated Syrian hamsters and the nasal turbinates and lungs of the contact female BALB/c mice.

To determine whether mice can be naturally infected by contact with other SARS-CoV-2-susceptible animals, we evaluated the direct contact transmission of the B.1.351 variant between Syrian hamsters and mice. Three male Syrian hamsters were inoculated with the virus on D0 and cohoused with six naïve female BALB/c mice on D1. Nasal washes were collected from the inoculated Syrian hamsters on D4, and the six contact BALB/c mice were sacrificed to collect nasal turbinates and lungs on D5. Then, the viral sgRNA load in the nasal washes of the inoculated Syrian hamsters and the nasal turbinates and lungs of the contact BALB/c mice was determined. Viral sgRNA was detected in the nasal washes of all inoculated Syrian hamsters, but no viral sgRNA was found in the nasal turbinates and lungs of the contact BALB/c mice (**Figure 4**). The B.1.351 variant was not transmitted from the inoculated Syrian hamster to naïve BALB/c mice.

## Discussion

In this study, we showed that the SARS-CoV-2 B.1.351 variant could infect mice and replicate efficiently in the airways of mice. This result is consistent with the results of two previous studies. Xavier Montagutelli et al. showed that the B1.351 variant replicated well in the lungs of BALB/c and C57BL/6 mice (11), and Qi Chen et al. showed that the B.1.351 variant infected and replicated to high titres in the lungs of aged female BALB/c mice (13). Additionally, we showed that the B.1.351 variant could infect rats and replicate moderately in the airways of rats. Huiping Shuai et al. also showed that SARS-CoV-2 variants with the N501Y mutation infected and replicated effectively in the airways of rats (18). An inoculation dose of 10^4^ TCID50 was used in all infected animals, which is a relatively high infection dose.

The SARS-CoV-2 receptor ACE2 proteins of Muridae are highly conserved, especially those critical amino acids involved in SARS-CoV-2 spike protein binding (**Supplementary Figure 2**). Several other species of Muridae can be infected by B.1.351, P.1 and other variants. Further molecular and serological investigation of SARS-CoV-2 variants in additional animals of the family Muridae is needed along the evolution of the virus.

In studying the emergence of new SARS-CoV-2 VOCs, in particular the N501Y variants, it was hypothesized that mice are potential transmitters of SARS-CoV-2 variants (17). We first evaluated the contact transmission of the B.1.351 variant in female BALB/c and C57BL/6 mice. Although the B.1.351 variant replicated efficiently in the nasal turbinates and lungs of the inoculated female BALB/c and C57BL/6 mice, no viral sgRNA was detected in the nasal turbinates or lungs of those mice in the contact transmission group. Generally, male mice are much more susceptible to SARS-CoV-2 than female mice, and the virus replicates more efficiently in male mice (14). Hence, we further evaluated the contact transmission of the B.1.351 variant in male BALB/c and C57BL/6 mice, but no viral sgRNA was detected in the nasal turbinates or lungs of male mice in the contact transmission group. In summary, the B.1.351 variant has not acquired the ability to transmit in mice. Additionally, we assessed the transmission ability of the mouse-adapted SARS-CoV-2 strain C57MA14 in six-week-old BALB/c mice (19). The C57MA14 virus can replicate efficiently in the airways of female and male BALB/c mice, but no viral sgRNA was detected in the airway samples of any BALB/c mice in the transmission group (**Supplementary Figure 3**). Hence, the mouse-adapted C57MA14 virus could not transmit in BALB/c mice.

We also evaluated the contact transmission of the B.1.351 variant in rats. The B.1.351 variant replicated moderately in the nasal turbinates and lungs of the inoculated rats, and a high level of viral sgRNA was detected in the nasal turbinates of three rats in the contact transmission group, but no viral sgRNA was detected in the lungs of rats in the transmission group. The B.1.351 variant has acquired moderate ability to transmit in rats by direct contact. Overall, the B.1.351 variant did not transmit in mice but could transmit in rats by direct contact. Previous studies have shown that SARS-CoV-2 could infect, replicate and transmit in minks and spillback to humans (20–22). At present, the possibility of mice as new reservoirs in the regions where the B.1.351 variant circulates should be extremely low, and the possibility of retrotransmission of B.1.351 and other variants from mice to humans is also extremely low. However, this is not true for rats. The B.1.351 variant can infect, replicate, and transmit in rats with moderate efficiency and is likely to establish effective infection chains. The transmission efficiency of the B.1.351 variant between rats is relatively low (50%) compared with transmission reported 100% in minks (20). We speculate that rats might be potential intermediate reservoirs of SARS-CoV-2. A recent study also proposed that rodents are the intermediate reservoirs of SARS-CoV-2 and SARS-CoV (23). To determine the potential prevalence of SARS-CoV-2 in rats, further serology and molecular surveillance of SARS-CoV-2 variants in domestic rats and wild rats should be performed in regions where B.1.351 and other variants circulate.

The Syrian hamster is a good small animal model to study SARS-CoV-2 transmission (24). Compared with that in the airways of mice, the B.1.351 variant replicated to higher titres in the airways of Syrian hamsters (25, 26). A high viral sgRNA load was also detected in the nasal washes of experimentally infected Syrian hamsters in our study. The inability of the B.1.351 variant to transmit in mice might be due to the relatively lower replication in the airways of mice, particularly in nasal turbinates. A previous study showed that viral replication in the upper respiratory tract rather than in the trachea and lower airways of donor ferrets is a driver of the transmission of influenza A viruses by aerosols (27). To determine whether mice can be naturally infected by exposure to high levels of viral excretion, we evaluated the contact transmission of the B.1.351 variant from Syrian hamsters to naïve BALB/c mice, but the B.1.351 variant was not transmitted from the inoculated Syrian hamsters to BALB/c mice. Therefore, the viral load differences in the airways do not explain the inability of B.1.351 transmission in mice. The underlying reason for the inability of B.1.351 to transmit in mice should be further explored.

In conclusion, the SARS-CoV-2 B.1.351 variant could infect mice and rats and replicate well in the airways of mice and rats. The SARS-CoV-2 B.1.351 variant has not acquired the ability to transmit in an experimental mouse model but did transmit in an experimental rat model by direct contact. Further evidence is needed to confirm SARS-CoV-2 transmission in wild rats but not in wild mice. At present, the risk of mice serving as new reservoirs for SARS-CoV-2 variants is extremely low, and the possibility of retrotransmission of B.1.351 from mice to humans is also extremely low. However, the risk of rats serving as new reservoirs for SARS-CoV-2 variants should not be neglected, and more attention should be given to the potential natural transmission of SARS-CoV-2 variants in and from domestic rats and wild rats. Continued surveillance of the evolution of SARS-CoV-2 and host range expansion remains necessary.

## Data Availability Statement

The original contributions presented in the study are included in the article/**Supplementary Material**, further inquiries can be directed to the corresponding author/s. 

## Ethics Statement

The animal study was reviewed and approved by Changchun Veterinary Research Institute.

## Author Contributions

CMZ, YG, and JL designed the project. CMZ, CZ, HC, EL, ZG, TW, LL, NL, FY, KM, YL, and DC performed the experiments and analyzed the data. CMZ drafted the manuscript, and YG, JL, and CQ critically revised the manuscript. All authors contributed to the article and approved the submitted version.

## Funding

This research was supported by the National Natural Science Foundation of China (32000134) and the National Major Research & Development Program (2020YFC0840800).

## Conflict of Interest

The authors declare that the research was conducted in the absence of any commercial or financial relationships that could be construed as a potential conflict of interest.

## Publisher’s Note

All claims expressed in this article are solely those of the authors and do not necessarily represent those of their affiliated organizations, or those of the publisher, the editors and the reviewers. Any product that may be evaluated in this article, or claim that may be made by its manufacturer, is not guaranteed or endorsed by the publisher.
