# Supplementary material for: The SARS-CoV-2 B.1.351 Variant Can Transmit in Rats But Not in Mice
Source: Front Immunol. 2022 Apr 28;13:869809. doi: 10.3389/fimmu.2022.869809 (PMC9095975; doi:10.3389/fimmu.2022.869809)

The SARS-CoV-2 B.1.351 Variant CanTransmit in Rats but not in Mice

Cheng Zhang^1, 2&^, Huan Cui^1, 3&^, Entao Li^1&^, Zhendong Guo^1^, Tiecheng Wang^1^, Fang Yan^1^, Lina Liu^1^, Yuanguo Li^1^, Di Chen^1^, Keyin Meng^1^, Nan Li^1^, Chengfeng Qin^4^, Juxiang Liu^2*^, Yuwei Gao^1*^, Chunmao Zhang^1*^

^1^ Changchun Veterinary Research Institute,Changchun, China

^2^ College of Veterinary Medicine, Hebei Agricultural University, Baoding, China

^3^ College of Veterinary Medicine, Jilin University, Changchun, China

^4^ Beijing Institute of Microbiology and Epidemiology, Beijing, China

^&^These authors contributed equally to this work.

*Corresponding authors:

Chunmao Zhang: [jk704715@sina.com](mailto:jk704715@sina.com)

Yuwei Gao: [gaoyuwei@gmail.com](mailto:gaoyuwei@gmail.com)

JuxiangLiu:ljx0315@126.com

**Materials and Methods**

**Ethics and biosecurity statement**

All animal experiments wereapproved by the Animal Care and Use Committee of Changchun Veterinary Research Institute (approval number: SMKX-20200915–11). All experiments involving infectious SARS-CoV-2 were performed atthe Animal Biosafety Level 3 Laboratories of Changchun Veterinary Research Institute.

**Cells, viruses and animals**

Vero-E6 cells (CRL1586, ATCC, USA) were grown in high-glucose Dulbecco’s modified Eagle’s medium (DMEM; HyClone, USA) supplemented with 10% foetal bovine serum (FBS; HyClone, USA), 100 U/mL penicillin and 100 μg/mL streptomycin at 37 °C with 5% CO_2_. The B.1.351 variant GDPCC (CSTR:16698.06.NPRC2.062100001) was isolated from an imported COVID-19 case from South Africa and propagated in Vero-E6 cells. C57MA14 is a mouse-adapted SARS-CoV-2 strain and was also propagated in Vero-E6 cells. The propagated virus was titrated by a TCID50 assay based on virus-induced cytopathic effects on Vero-E6 cells. Six-week-old BALB/c and C57BL/6 mice and eight-week-old rats were purchased from Liaoning Changsheng Biotechnology Co., Ltd. During the challenge and transmission experiments, all animals were kept in independent ventilated cages in Animal Biosafety Level 3 Laboratories.

**Viral replication in mice and rats**

To evaluate viral replication in vivo, six female BALB/c and six female C57BL/6 mice were anaesthetized with isoflurane and intranasally inoculated with 2×10^4^TCID_50_ of the B.1.351 variant in 50 μL of PBS, and six male rats were anaesthetized and intranasally inoculated with 2×10^4^TCID_50_ of the virus in 200 μL of PBS on day 0 (D0). On D2 and D4, three BALB/c or C57BL/6mice and rats were sacrificed to collect the nasal turbinates and lungs. The collected tissues were homogenized in 1 mL of PBS with a tissue homogenizer, and the centrifuged tissue supernatants were used for viral subgenomic RNA (sgRNA) quantification and viral titre determination.

**Viral transmission study**

To evaluate viral transmission between mice by direct contact, six female BALB/c mice were anaesthetized and inoculated with 2×10^4^TCID_50_ of the B.1.351 variant on D0, and another six naïve female BALB/c mice were transferred to a new cage and cohoused with the six inoculated BALB/c mice on D1. The inoculated BALB/c and the contact BALB/c were sacrificed to collect the nasal turbinates and lungs on D4 and D5 respectively. The collected tissues were homogenized in 1 mL of PBS with a tissue homogenizer, and the centrifuged tissue supernatants were used for viral subgenomic RNA quantification. Similar to female BALB/c mice, six inoculated female C57BL/6 mice and six naïve female C57BL/6 mice were also used for the viral transmission study. To evaluate the sex differences on viral transmission, twelve male BALB/c and twelve male C57BL/6 mice were also used for the viral transmission study.

To evaluate the viral transmission between rats by direct contact, three groups of two male rats were anaesthetized and inoculated with 2×10^4^TCID_50_ of the B.1.351 variant in 200 μLof PBS on D0, and another three groups of two naïve male rats were paired with the three groups of inoculated rats, transferred to three new cages and cohoused together (donor-contact ratio: 2:2) on D1. The inoculated rats and the contact rats were sacrificed to collect the nasal turbinates and lungs on D4 and D5 respectively. The homogenized tissue supernatants were used for viral subgenomic RNA quantification.

To evaluate the viral transmission between Syrian hamsters and mice by direct contact, three male Syrian hamsters were anaesthetized and inoculated with 2×10^4^TCID_50_ of the B.1.351 variant in 100 μL of PBS on D0, and six naïve female BALB/c mice were transferred to a new cage and cohoused together with the three inoculated donor hamsters on D1. Nasal washes were collected from all inoculated hamsters using 1 mL of PBS on D4, and the six contact female BALB/c mice were sacrificed to collect the nasal turbinates and lungs on D5. The homogenized tissue supernatants were used for viral subgenomic RNA quantification.

To evaluate the contact transmission of the mouse-adapted C57MA14 virus between mice, six female or male BALB/c mice were inoculated with 500 TCID_50_ of the virus on D0, and then the experiment was performed similarly to the transmission experiments of the B.1.351 variant in mice.

**Viral subgenomic RNA quantification**

The viral sgRNA load was determined as described previously, with a slight modification([1](#_ENREF_1)). RNA was extracted from 200 μL of the homogenized tissue supernatants using viral RNA Mini Kits (QIAGEN, Hilden, Germany) and eluted with 90 μL of distilled water. For sgRNA quantification, a pair of primersand a TaqMan probe were designed targeting the E genesgRNA of SARS-CoV-2([2](#_ENREF_2)), and 9 μLof RNA was used for real-time qPCR using One Step PrimeScript^TM^ III RT–qPCR Mix (catalogue No: RR600A, Takara). The qPCRs were performed as described previously([1](#_ENREF_1)). The number of sgRNA copies in each sample was estimated from the measured cycle threshold (Ct) based on an established standard curve. The standard curve was fitted using a series of 10-fold dilutions of a standard plasmid of the E gene of SARS-CoV-2. The fitted standard curve equation was Ct = -3.11X0 + 41.16, where X0 is the initial viral RNA copy number in the reaction system.

**Statistical analysis**

Anunpaired t test was used to analyse the significant differences in viral titres and viral sgRNA loads between the nasal turbinates and lungs (p<0.05,*; p<0.01,**; p<0.001,***). The data are presented as the mean ± sem values. The numbers of replicate animals (n) are stated in the figure legends.Statistical analyses were carried out using Prism software (GraphPad Prism 6.01).

**References**

1. Zhang C, Guo Z, Li N, Cui H, Meng K, Liu L, et al. Impact of Prior Infection on Severe Acute Respiratory Syndrome Coronavirus 2 Transmission in Syrian Hamsters. *Frontiers in microbiology* (2021) 12:722178. doi: 10.3389/fmicb.2021.722178.

2. Wolfel R, Corman VM, Guggemos W, Seilmaier M, Zange S, Muller MA, et al. Virological Assessment of Hospitalized Patients with Covid-2019. *Nature* (2020) 581(7809):465-9. doi: 10.1038/s41586-020-2196-x.

**Figure Legends**

**Supplementary Figure 1**SARS-CoV-2 B.1.351 variant transmission in male BALB/c and male C57BL/6 mice. The dotted line represents the detection limit. Viral sgRNA copies (log_10_ sgRNA copies/mL) in the nasal turbinates (A) and lungs (B) of inoculated male BALB/c mice and contact male BALB/c mice. Viral sgRNA copies in the nasal turbinates (C) and lungs (D) of inoculated male C57BL/6 mice and contact male C57BL/6 mice.


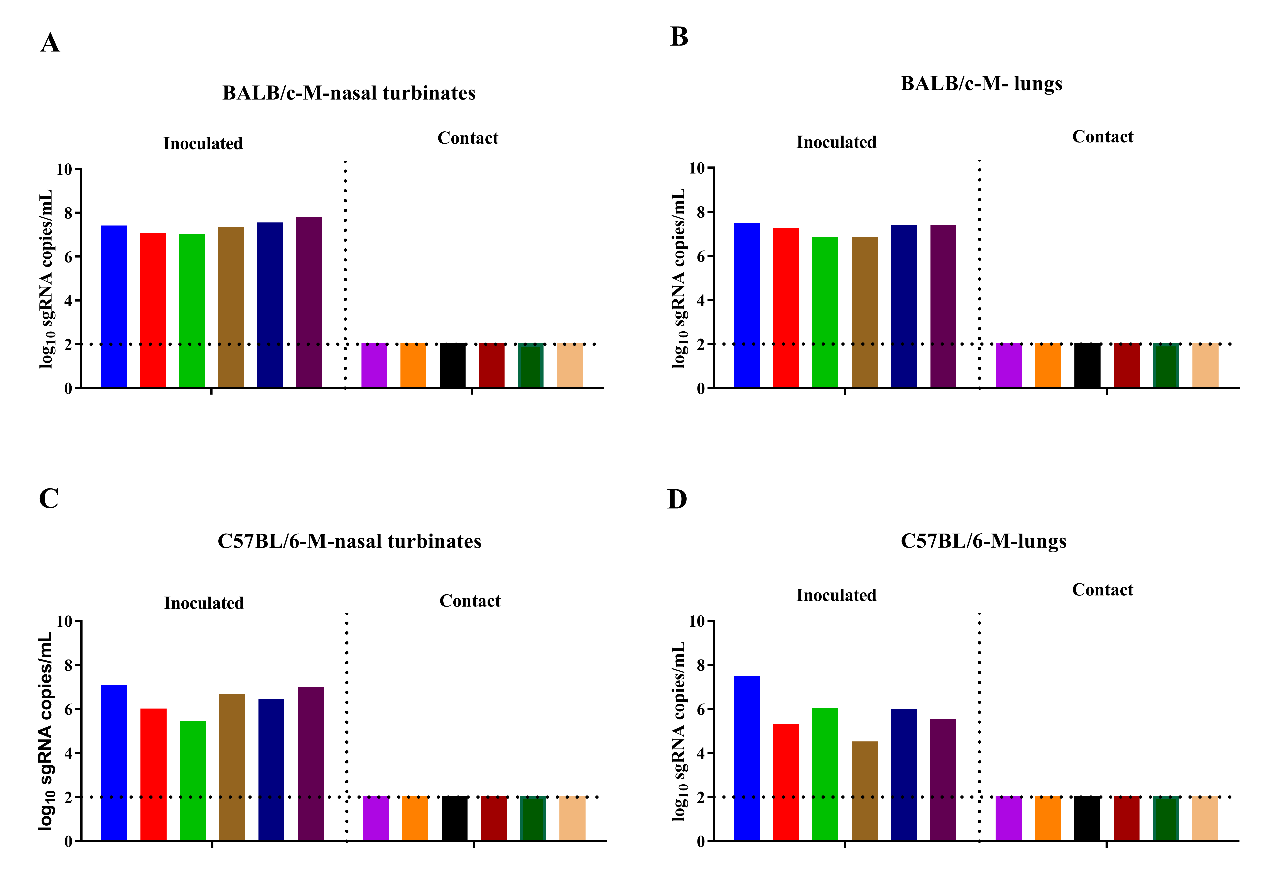


**Supplementary Figure 2** Amino acid sequence alignment of ACE2 proteins from animals of the Muridae family. Those key amino acids associated with the binding of the receptor-binding domain of the spike protein arelabelled with* beneath the letter.


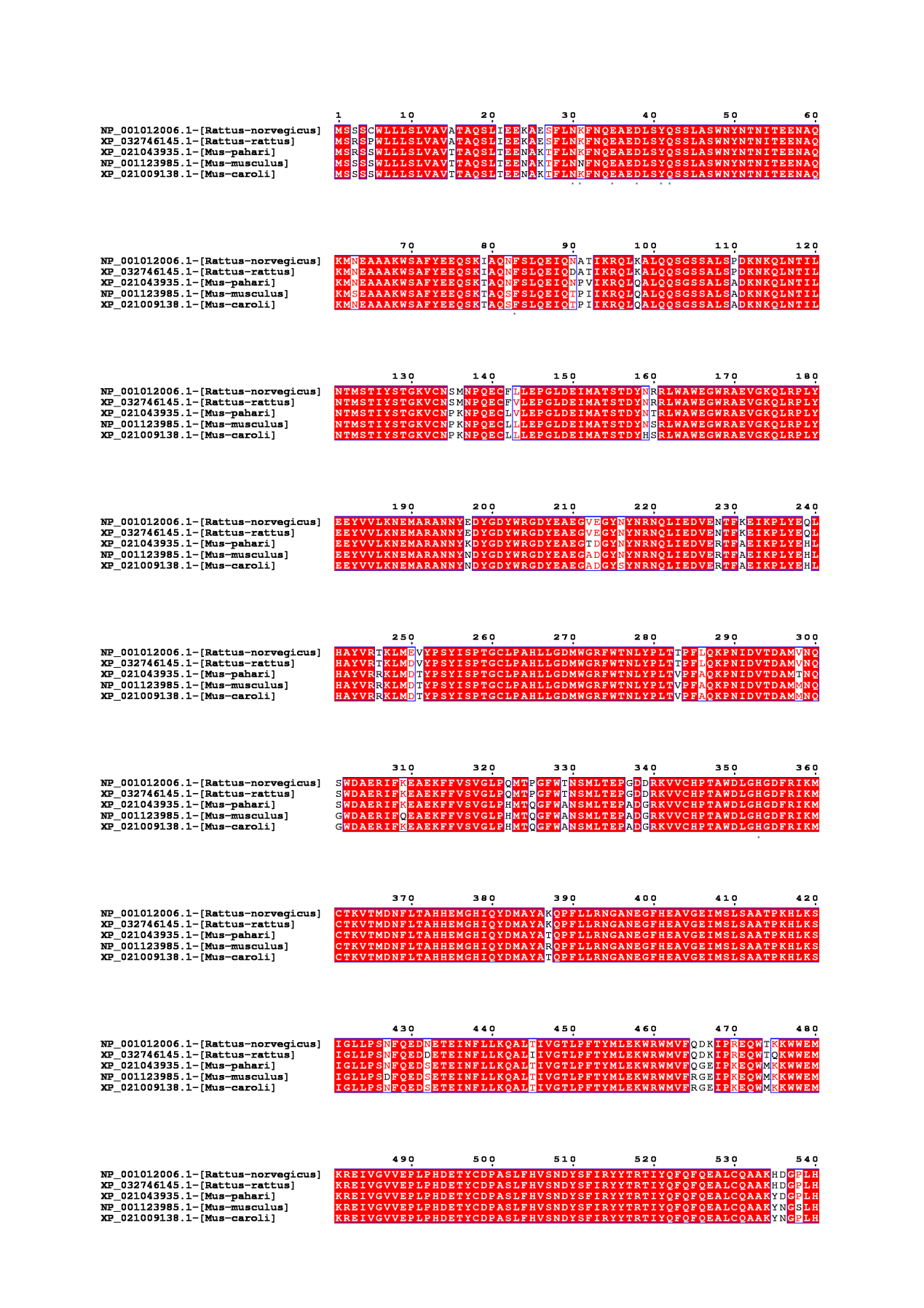


**
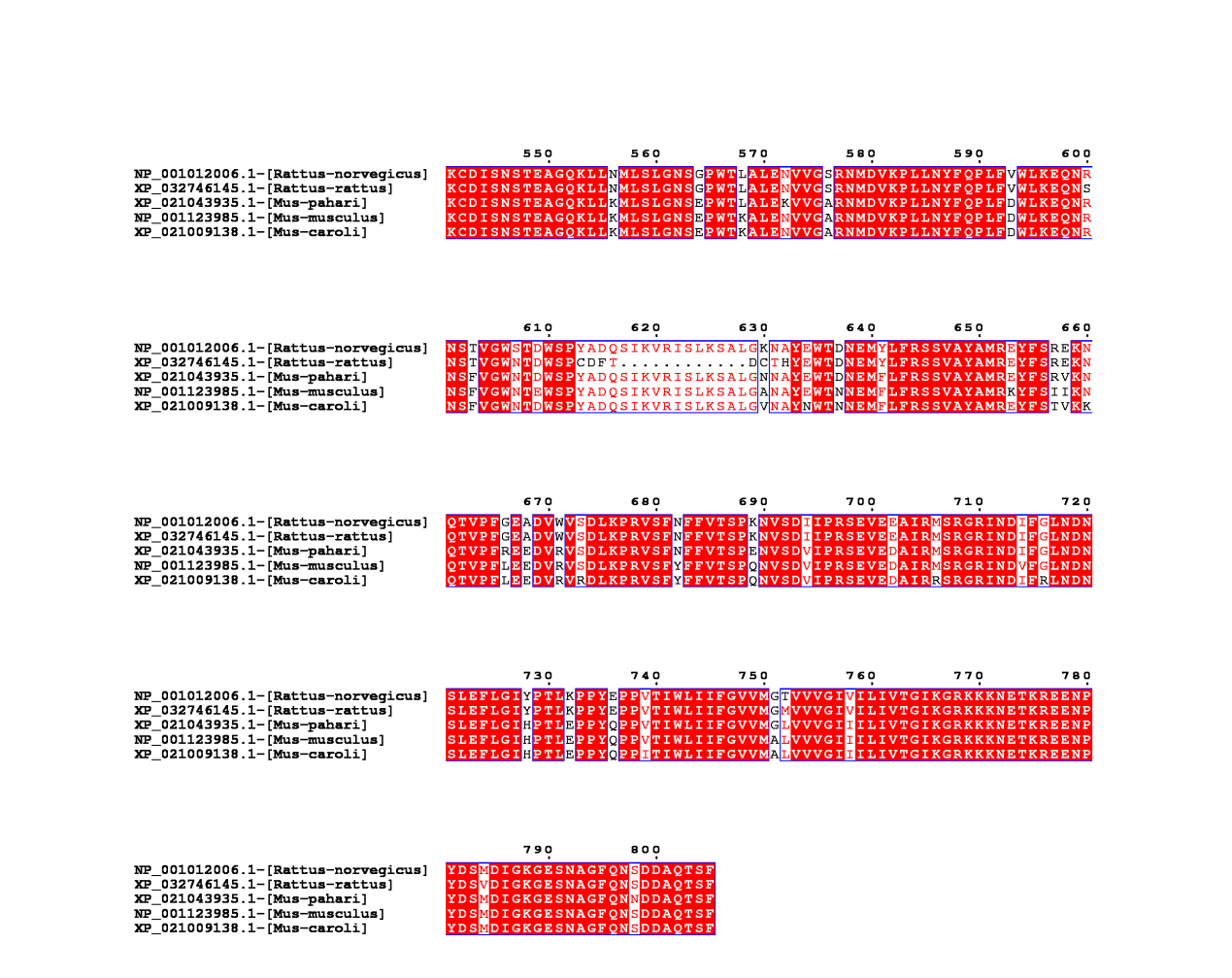
**

**Supplementary Figure 3**Mouse-adapted SARS-CoV-2 strain CMA14 transmission in BALB/c mice. The dotted line represents the detection limit. Viral sgRNA copies (log_10_ sgRNA copies/mL) in the nasal turbinates (A) and lungs (B) of the inoculated female BALB/c mice and the contact female BALB/c mice. Viral sgRNA copies in the nasal turbinates (C) and lungs (D) of inoculated male BALB/c mice and contact male BALB/c mice.


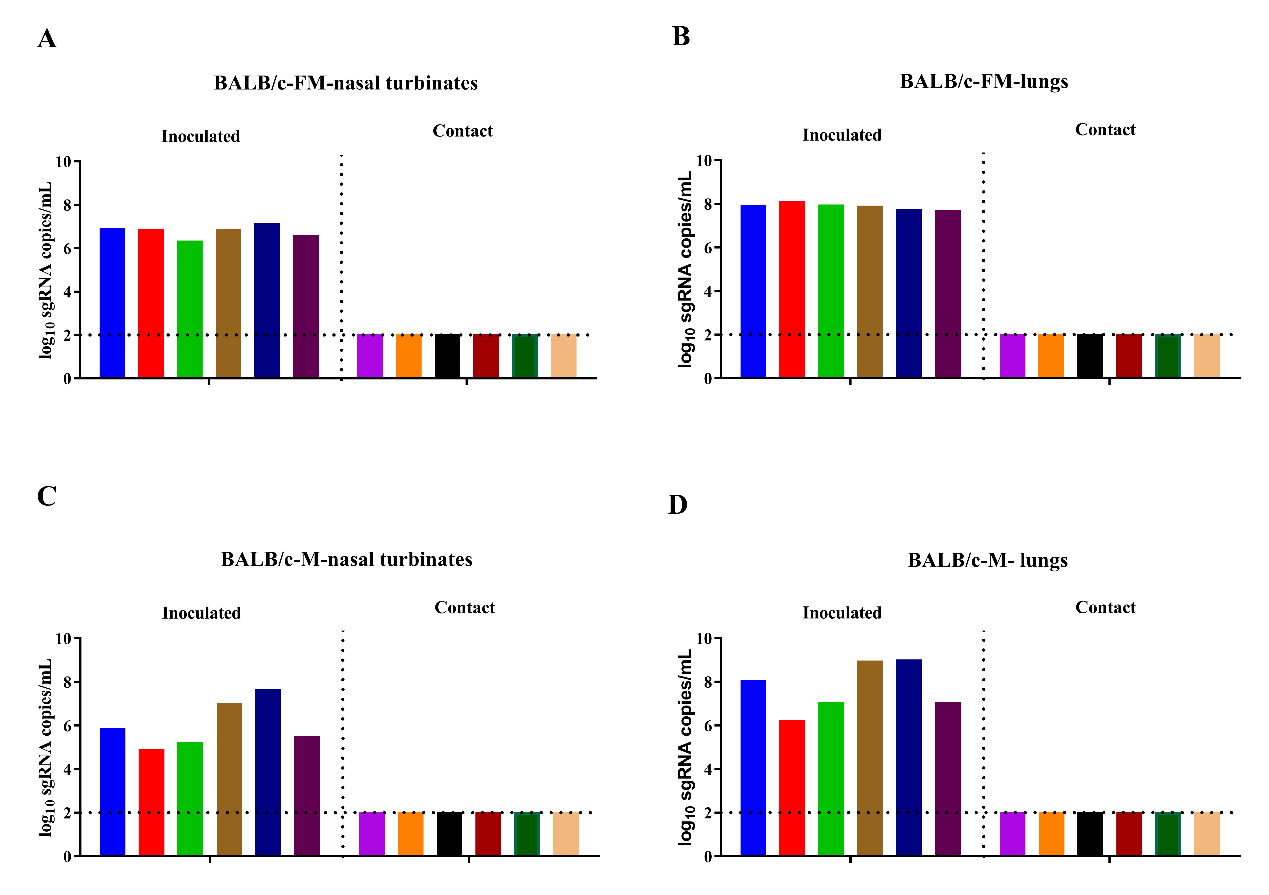

Supplement: Supplementary file 1 [file DataSheet_1.docx]
